# Noncausal effects of genetic predicted depression and colorectal cancer risk: A Mendelian randomization study

**DOI:** 10.1097/MD.0000000000030177

**Published:** 2022-08-26

**Authors:** E. Wu, Jun-Tao Ni, Tian Xie, Lin Tao

**Affiliations:** a School of Pharmacy, Hangzhou Normal University, Hangzhou, China; b Key Laboratory of Elemene Class Anti-Cancer Chinese Medicines, Engineering Laboratory of Development and Application of Traditional Chinese Medicines, Collaborative Innovation Center of Traditional Chinese Medicines of Zhejiang Province, Hangzhou Normal University, Hangzhou, China; c Women’s Hospital, School of Medicine, Zhejiang University, Hangzhou, China.

**Keywords:** causal relationship, colorectal cancer, depression, Mendelian randomization, prevention

## Abstract

Depression has been associated with colorectal cancer (CRC) in observational studies. However, the causality of depression on CRC risk remained unknown. This study aimed to evaluate the potential causal association between genetic variants related to depression and the risk of CRC using Mendelian randomization (MR). Two-sample MR analysis using summary data was performed to examine whether depression was causally associated with CRC risk. We used 2 sets of instrumental variables (IV) from the genome-wide association study results for analysis. A set of IV related to major depressive disorder contain 44 single-nucleotide polymorphisms. Another set of IV was related to major depression, including 53 single-nucleotide polymorphisms. Summary data of CRC was from the FinnGen consortium. Based on the results of MR using inverse-variance weighted method, we found that genetically determined major depressive disorder (odds ratio = 1.06, 95% confidence interval = 0.77–1.45) or major depression (odds ratio = 0.77, 95% confidence interval = 0.57–1.04) did not causally increase CRC risk. The results of MR-Egger and the weighted median method are consistent with the inverse-variance weighted method. The two-sample MR analysis showed that depression is not causally associated with CRC risk. Further research is needed to investigate the association between depression and CRC.

## 1. Introduction

Colorectal cancer (CRC) is the third most common cancer and the second leading cause of cancer-related death in the world, with an estimate of nearly 2 million new cases and approximately 1 million deaths worldwide in 2018, which will have a significant impact on national health and social services.^[1–3]^ Known risk factors related to CRC included gender, advanced age, family history, genetic, and racial factors, as well as modifiable risk factors, such as obesity, drinking, smoking, poor exercise habits, and psychiatric conditions.^[4,5]^ To further reduce the economic and social burden caused by CRC, it is imperative to identify the modifiable determinants for CRC prevention.

Depression is a common and treatable mental disorder, with an estimate of 270 million people worldwide suffering from depression in 2019.^[6]^ It is a modifiable psychiatric condition that has emerged as a potential risk factor for various diseases, and a recent review article indicated that depression was highly related to cancers, such as breast, pancreatic, oropharyngeal, lung, and CRCs.^[7]^ Notably, an observational study found that depression can not only be associated with CRC but may also be a sign of accelerated CRC progression, with higher depression symptoms associated with greater CRC-related mortality risk (hazard ratio: 1.17, 95% confidence interval [CI]: 1.01–1.34).^[8]^ However, it remains unclear whether depression may be causally related to CRC risk due to the observational studies being limited by potential biases of confounding or reverse causality. Under the circumstance without a randomized controlled trial (RCT), 2-sample Mendelian randomization (MR) emerges as a novel method for examination of causal affection of exposures in observational studies.^[9]^

MR analysis uses genetic variants as instrumental variables (IV) to deduce the causal affection between possible risk factors and explicit outcomes.^[10]^ It is similar to a “hereditary RCT” due to MR based on Mendel law of random allocation. That is, the alleles of gametes follow the principle of random allocation in the process of meiosis, which can effectively avoid the influence of measurement errors, confounding bias, and reverse causality in observation studies^.[11]^ Hence, genetic variants associated with depression could serve as IV to examine the correlation of depression with CRC risk. In this study, we conducted a 2-sample MR method to assess the causal effect of depression on the risk of CRC.

## 2. Methods

### 2.1. Study design

**Figure 1.**
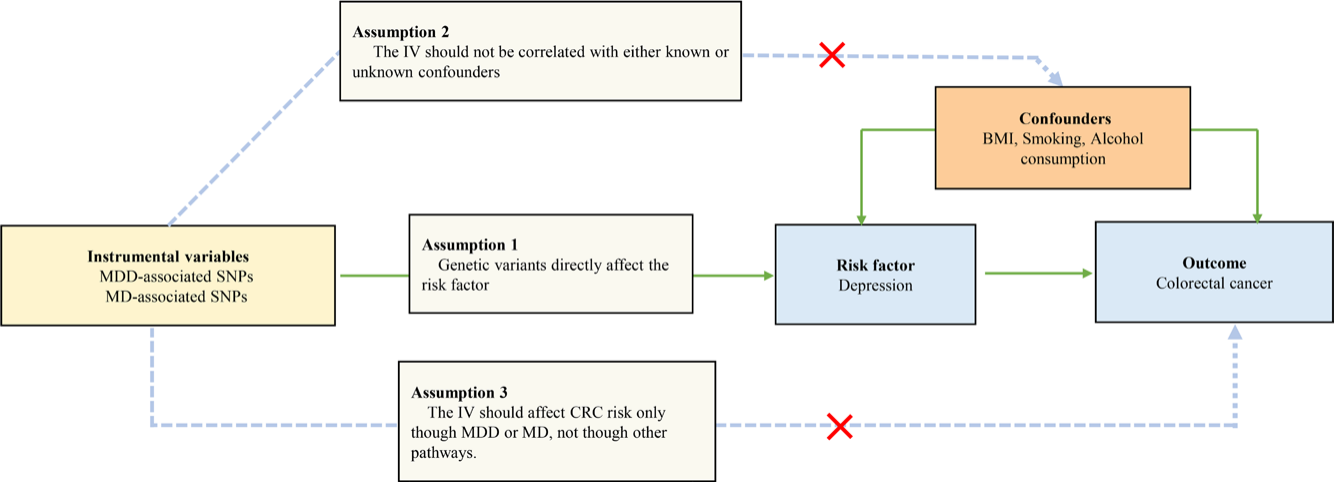
Schematic diagram of the MR assumptions. CRC = colorectal cancer, IV = instrumental variables, MD = major depression, MDD = major depressive disorder.

The 2-sample MR approach in this study is based on 3 principal assumptions (Fig. 1). Firstly, the genetic variants as IV should relate to depression. To avoid selection bias caused by a single set of IV, we use 2 sets of IV, which genetic variants related to major depressive disorder (MDD) or major depression (MD), to examine the causality of depression on CRC. Secondly, the IV should not be correlated with potential confounders. Thirdly, the IV should affect CRC risk only through MDD or MD, not through other pathways. Notably, in 2-sample MR, the summary data related to single-nucleotide polymorphism (SNP)-exposure and SNP-outcome should come from different research sources, which means the research objects should not overlap.^[12]^

### 2.2. Genome-wide association study summary data on MDD

Genome-wide association study (GWAS) identified 44 SNPs associated with MDD in a meta-analysis of 480,359 individuals of European ancestry (*P* < 5.0 × 10^−8^), with 344,901 controls and 135,458 MDD cases from various countries such as USA, Australia, UK, the Netherlands, Germany, Italy, Sweden, Iceland, Switzerland, and Denmark.^[13]^ The *F*-statistics for the 44 SNPs was 156, which revealed sufficient instrument strength for analyses.^[14]^ Five SNPs with *r*^2^ > 0.01 of linkage disequilibrium in the range of 5000 kb were excluded. Another 3 SNPs with palindromic were further removed. The rest 36 SNPs will be further matched with CRC summary data.

### 2.3. GWAS summary data on MD

To avoid inconsistent results caused by the selection bias of SNPs variants associated with depression, we built another set of IV which related to MD, from a GWAS meta-analysis, which includes 329,443 controls and 170,756 MD cases of European ancestry. After removing SNPs with linkage disequilibrium (*r*^2^ > 0.01) in the range of 5000 kb, 53 SNPs associated with MD from UK Biobank and Psychiatric Genomics Consortium (excluding “23andme”) were obtained (*P* < 5.0 × 10^−8^).^[15]^ We further excluded 9 SNPs with palindromic. *F*-statistics for the rest 44 SNPs was 179, indicating that the IV had great potential for MR analyses.

### 2.4. GWAS summary data on CRC

Summary data for CRC was obtained from the FinnGen consortium (https://finngen.gitbook.io/documentation). Totally, there were 218,792 individuals of Finn, with 215,770 controls and 3022 CRC cases. We retrieved GWAS summary data from FinnGen consortium. Each of the 36 SNPs related to MDD and 41 SNPs related to MD was extracted. The details of the 36 SNPs associated with MDD and 41 SNPs associated with MD were listed in Table S1 (Supplemental Digital Content, http://links.lww.com/MD/H68) and Table S2 (Supplemental Digital Content, http://links.lww.com/MD/H71), respectively.

### 2.5. GWAS summary data for confounders

To avoid the mediating effect of MDD or MD on CRC via confounders, we performed inverse-variance weighted (IVW) method to examine the correlations between MDD or MD and well-accepted confounding factors, including drinking, smoking, and body mass index (BMI).^[16,17]^ GWAS summary data for these confounders were obtained from the meta-analysis of GWAS and FinnGen consortium. The details of all GWAS summary data included in this research are shown in Table S3 (Supplemental Digital Content, http://links.lww.com/MD/H72).

### 2.6. Statistical analyses

SNPs were harmonized by assigning to the same effect allele across all summary data of this study. SNPs with palindrome structures were further removed. An IVW approach was performed to present an overall estimate of causal effects. Its characteristic is that the regression does not consider the existence of the intercept term and uses the reciprocal of the outcome variance (the square of standard error) as the weight for modeling. When using the IVW method, it is necessary to ensure that these SNPs are valid IV and without horizontal pleiotropy. Otherwise, the results will be biased. In order to avoid violating the assumption of IV and the existence of horizontal pleiotropy, we performed a weighted median and MR-Egger method to complement the IVW MR analysis. Due to a weighted median method that allows half of the IV to be invalid,^[18]^ the MR-Egger method allows the existence of horizontal pleiotropy.^[9]^ Sensitivity analysis of the MR results was performed from 3 aspects, including the heterogeneity test, pleiotropy test, and leave-one-out sensitivity test.

MR pleiotropy residual sum and outlier (MR-PRESSO) method was performed to test and correct horizontal pleiotropic outliers.^[19]^ MR-PRESSO contains 3 parts. First, the global test of heterogeneity was conducted to test horizontal pleiotropy. Second, the outlier test identified outliers (*P* < .05), and the detected outliers will be removed to give a correction result. Third, the distortion test was performed to check the significant difference prior to and after outliers removal correction.

Analyses and graphic plotting were performed using packages of 2 Sample MR (version 0.5.6) and MR-PRESSO (version 1.0) in R (version 4.1.0; R Core Team [2021]; R Foundation for Statistical Computing; Vienna, Austria). All *P* values were 2-sided, and *P* value of <.05 was considered statistically significant.

### 2.7. Ethical approval

The MR approach in this study is based on publicly available GWAS summary data. Therefore, additional ethical approval was not required.

## 3. Results

We harmonized SNPs of MDD-CRC and MD-CRC datasets and identified 36 SNPs significantly associated with MDD and 41 SNPs significantly associated with MD that could also be extracted from summary data of CRC, all of which meet the conditions of MR analysis.

**Figure 2.**
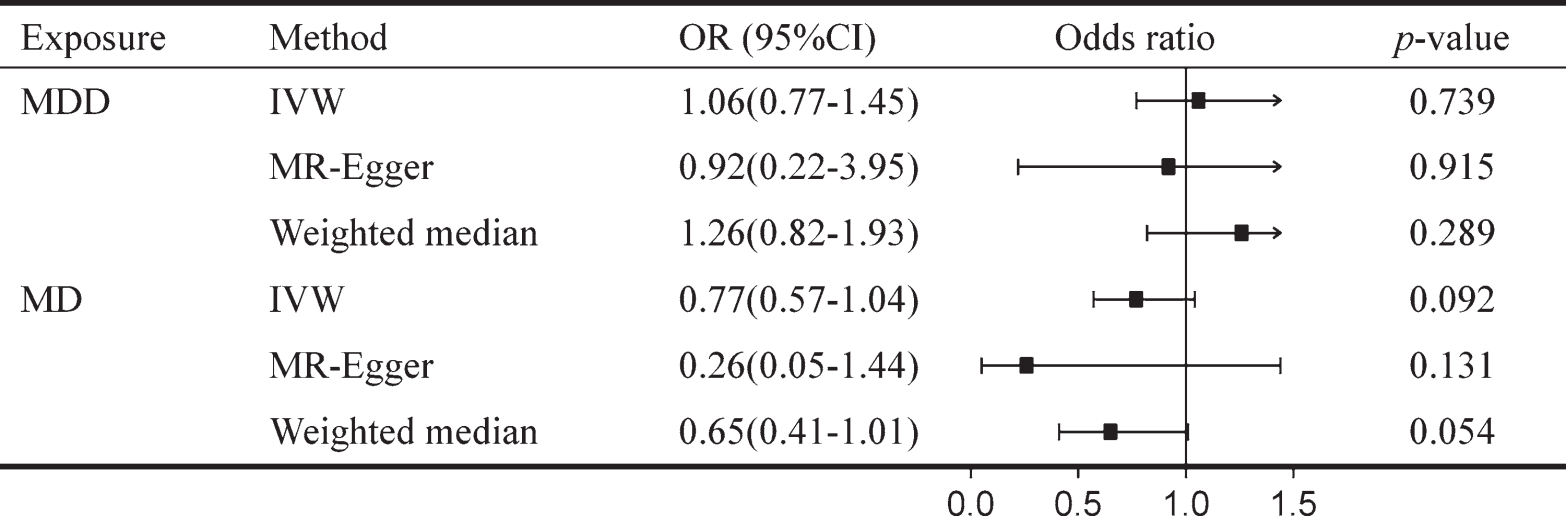
MR estimates of the associations between MDD and MD on CRC. IVW = inverse variance weighted method, MD = major depression, MDD = major depressive disorder.

**Figure 3.**
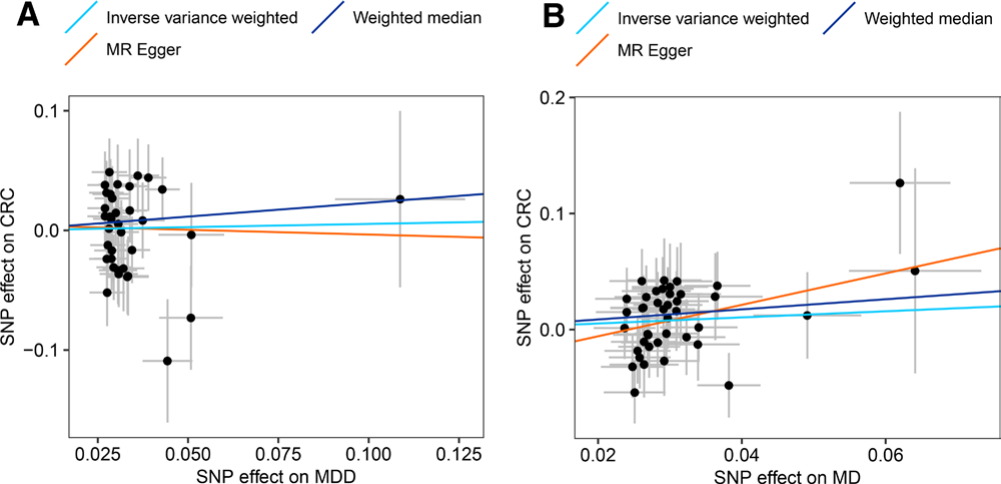
Scatter plot of SNPs related to MDD or MD and the CRC risk. (A) Scatter plot of MDD-CRC risk MR. (B) Scatter plot of MD-CRC risk MR. CRC = colorectal cancer, MD = major depression, MDD = major depressive disorder, SNP = single-nucleotide polymorphism.

According to the IVW method, no significant causal effect was observed between MDD and MD with CRC risk. Odds ratio (OR) of MDD and MD were 1.06 (95% CI = 0.77–1.45) and 0.77 (95% CI = 0.57–1.04), respectively (Fig. 2). Results of using the MR-Egger method and weighted median method were consistent, although MR-Egger regression presented a wider CI (*P* > .05). Fig. 3 showed the scatter plots of association estimates between SNPs associated with MDD or MD and SNPs related to CRC, as well as the MR causal estimates. The forest plot of SNPs related to MDD or MD and their CRC risk was shown in Figure S1 (Supplemental Digital Content, http://links.lww.com/MD/H73; *P* > .05). The MR-PRESSO and MR-Egger analysis showed no horizontal pleiotropy between MDD or MD and CRC, and no heterogeneity between the MR estimates from different SNPs has been observed (*P* > .05; Table S4, Supplemental Digital Content, http://links.lww.com/MD/H74). Furthermore, the leave-one-out sensitivity test (Figure S2, Supplemental Digital Content, http://links.lww.com/MD/H75) and the funnel plot (Figure S3, Supplemental Digital Content, http://links.lww.com/MD/H76) indicated that the MR result was reliable for the IV.

To test the causal estimates between genetically determined MDD or MD and CRC risk was not through the pleiotropic pathways of depression-CRC related confounders, such as smoking, alcohol consumption, and BMI. As shown in Table 1, using IVW, genetically predicted MDD was not causally associated with smoking (OR = 1.21, 95% CI = 0.78–1.89), alcohol consumption (OR = 0.96, 95% CI = 0.91–1.01), and BMI (OR = 1.00, 95% CI = 0.87–1.14). No causal correlation has been observed between MD and smoking (OR = 1.16, 95% CI = 0.73–1.85), alcohol consumption (OR = 0.96, 95% CI = 0.91–1.01), or BMI (OR = 1.11, 95% CI = 0.96–1.30).

**Table 1:** MR estimates of the associations from depression on common confounders.

Exposure	Confounder	SNPs (No.)	Outliers (No.)	OR (95%CI)	*P* value
MDD	Smoking	36	0	1.21 (0.78–1.89)	.386
	Alcohol consumption	35	6	0.96 (0.91–1.01)	.087
	BMI	13	1	1.00 (0.87–1.14)	.949
MD	Smoking	41	0	1.16 (0.73–1.85)	.525
	Alcohol consumption	41	4	0.96 (0.91–1.01)	.074
	BMI	25	4	1.11 (0.96–1.30)	.169

BMI = body mass index, CI = confidence interval, MD = major depression, MDD = major depressive disorder, OR = odds ratio, SNPs = single nucleotide polymorphisms.

## 4. Discussion

In this 2-sample MR study using 2 sets of IV and 3 complementary statistical methods, we found consistent evidence that genetically predicted depression did not causally influence the risk of CRC.

So far, the relationship between depression and CRC has not been clearly clarified. Even though the previous observational study indicated that depression was associated with CRC risk (OR = 1.37, 95% CI = 1.18–1.58),^[20]^ the previous epidemiological studies were mostly cross-sectional design, which cannot infer the causality due to the fuzzy temporal order. Besides, studies from prospective cohorts indicated that CRC patients seemed to be more vulnerable to depression.^[21]^ Thus, depression may not be a predictor of CRC development but may be a result of CRC. For MR, analyses seem to be like a genetic RCT due to genotypes being randomly assigned from parents to offspring, which may decrease confounding bias and reverse causality, making causal effects infer more powerfully. As such, our study offered a cautious explanation for depression promoting the onset or development of CRC, and further emphasized that future observational studies need to consider potential confounding factors and reverse causality.

Although depression may not directly affect the risk of CRC, it may have been implicated as a potential mediator in the progression of CRC. First, biological evidence suggests that high levels of depression may affect the sympathetic nervous system and the function of the hypothalamic–pituitary–adrenal axis; for depression, may include overactive hypothalamic–pituitary–adrenal axis, which manifests as an increase in corticotropin-releasing factor and corticotropin, leading to an increase in plasma and urinary-free cortisol levels, which may modify the immune response and inflammatory process involved in the process of CRC.^[22–24]^ Second, increases in inflammatory cytokines have been observed in patients with depression, such as increased levels of proinflammatory cytokines interleukin 1β, interleukin 6, interferon γ, and tumor necrosis factor α, and antiinflammatory cytokines IL-10 and IL-1 receptor antagonism release increased, as well as the nuclear factor kappa B binding increases, as nuclear factor kappa B is a vital signal molecule in the inflammatory cascade and has been identified with cancer development, which may promote inflammation, and eventually predisposes to cancer.^[25]^ Third, studies have found that immunosuppression in patients with depression is manifested by changes in cellular immune function and the number of immune cells, such as a decrease in the total number of lymphocytes, in which the percentage of CD3+, CD4+ cells and the ratio of CD4+/CD8+ decreases, while the percentage of CD8+ cells increases, and the activity of natural killer (NK) cells and DNA repair enzymes inhibited; the immunosuppression manifested by patients with depression weakens the tumor defense function of the immune system, which is conducive to the growth and proliferation of tumor cells.^[14]^ Fourth, several unhealthy lifestyle habits associated with depression, such as smoking, drinking, and antidepressants, may indirectly lead to CRC.^[5]^ In short, depression is a common mental abnormality, with complex and contradictory immune changes that may affect the occurrence, development, prognosis, and outcome of cancer through various factors, such as the neuroendocrine-immune regulatory system, but the exploration of its intrinsic biological mechanism is still in the initial stage. Therefore, the role of depression in the formation and development of CRC deserves further study.

Even though genetically predicted depression may not causally influence the CRC risk, it was relatively consistent that depression in CRC patients was associated with a poor prognosis.^[26–29]^ A prospective study from Claudia indicated that CRC individuals with depression present a high overall mortality risk. For every 1 standard deviation increase in depressive symptoms, the risk of death increases by 16% (95% CI = 1.07–1.26).^[8]^ Chida et al^[30]^ also found that depression was related to higher mortality in cancer patients (RR = 1.08). Several studies have reported that depression may partially lead to a prolonged hospital stay, decreased quality of life, and diminished adherence to treatment.^[31]^ Besides, reduction of depression symptoms through psychotherapy has been identified to be associated with the increasing number and activity of NK cells, as well as increased survival in several cancer patients, such as malignant melanoma and breast cancer.^[25]^ All in all, treatment of depression can not only improve the patient’s mood, but may also improve the CRC-related prognosis; it is therefore essential to treat patients’ depression.

The current study has several advantages. First, MR analysis seems to be less prone to the bias of observational research, particularly in terms of reverse causality and confounders, providing a more reliable estimate of the causal relationship between depression and CRC outcome. Second, this MR analysis includes the use of a large sample size and 2 sets of uncorrelated SNPs related to depression to avoid selection bias, as well as the complementary MR methods involved in this study, which improves the precision of the estimate.

The current study has several limitations that should be mentioned. First of all, our research is limited to participants of European ancestry, which does not necessarily apply to other ethnic groups. Secondly, even though we do use MR-Egger regression and MR-PRESSO to estimate the degree of heterogeneity and pleiotropy that may bias the results, we cannot be sure that the selected genetically predicted depression-associated SNPs do not violate the possibility of the MR hypothesis. Finally, our research did not pay attention to the specific types of CRC, such as ascending colon cancer, descending colon cancer, transverse colon cancer, rectal cancer, etc. Depression may be causally related to a certain kind of CRC, which needs extensive research in the future.

## 5. Conclusions

We found that genetically predicted depression was not causally associated with CRC risk. This finding implied that strengthening screening CRC in patients with depression may be useless. More attention should be focused on revealing the association between environment-determined depression and CRC or depression and prognosis of CRC.

## Author contributions

EW performed the statistical analysis and wrote the manuscript. J-TN designed the study. LT and TX revised the manuscript. All authors read and approved the final manuscript.

**Data curation:** E Wu.

**Formal analysis:** E W.u

**Funding acquisition:** Jun-Tao Ni, Lin Tao.

**Methodology:** Jun-Tao Ni.

**Writing – original draft:** E Wu.

**Writing – review & editing:** Lin Tao, Tian Xie.

## Supplementary Material


