# Supplementary material for: Noncausal effects of genetic predicted depression and colorectal cancer risk: A Mendelian randomization study
Source: Medicine (Baltimore). 2022 Aug 26;101(34):e30177. doi: 10.1097/MD.0000000000030177 (PMC9410676; doi:10.1097/MD.0000000000030177)
Supplement: Supplementary file 1 [file medi-101-e30177-s001.pdf]

Supplementary Table 1. 36 SNPs associated with MDD

SNP	CHR	Effect allele	Other allele	Beta	SE	P value	Sample size
rs1432639	1	A	C	0.039	0.005	4.55E-15	480359
rs9427672	1	G	A	0.0321	0.0058	3.12E-08	480359
rs4261101	1	A	G	-0.0285	0.005	1.04E-08	480359
rs159963	1	A	C	-0.027	0.0049	3.19E-08	480359
rs12129573	1	A	C	0.034498	0.005	4.01E-12	480359
rs2389016	1	T	C	0.0305	0.0053	1.02E-08	480359
rs1226412	2	T	C	0.033203	0.0059	2.38E-08	480359
rs11682175	2	C	T	0.028101	0.0048	4.68E-09	480359
rs76485002	2	G	A	-0.1087	0.018	1.60E-09	480359
rs7430565	3	A	G	-0.0288	0.0048	2.87E-09	480359
rs4869056	5	A	G	-0.0287	0.005	6.80E-09	480359
rs247910	5	G	A	0.031501	0.0049	1.07E-10	480359
rs11135349	5	C	A	0.029398	0.0048	1.09E-09	480359
rs6905391	6	A	G	-0.0443	0.0069	1.35E-10	480359
rs10950398	7	A	G	0.027498	0.0049	2.55E-08	480359
rs12666117	7	A	G	0.027401	0.0048	1.35E-08	480359
rs10959913	9	G	T	-0.0334	0.0057	5.06E-09	480359
rs7856424	9	T	C	-0.0306	0.0053	8.48E-09	480359
rs1354115	9	A	C	0.027596	0.0049	2.37E-08	480359
rs61867293	10	T	C	-0.0374	0.0061	6.97E-10	480359
rs1806153	11	T	G	0.036101	0.0059	1.18E-09	480359
rs4074723	12	C	A	0.027001	0.0049	3.12E-08	480359
rs4143229	13	A	C	-0.0509	0.0091	2.51E-08	480359
rs12552	13	G	A	-0.0429	0.0048	6.07E-19	480359

rs915057	14	G	A	0.029995	0.0049	7.61E-10	480359
rs2005864	14	T	C	0.028199	0.0049	6.73E-09	480359
rs10149470	14	G	A	0.028996	0.0049	3.05E-09	480359
rs4904738	14	C	T	0.028904	0.0049	2.57E-09	480359
rs8025231	15	C	A	0.033898	0.0048	2.36E-12	480359
rs7200826	16	T	C	0.030704	0.0055	2.43E-08	480359
rs7198928	16	C	T	-0.0284	0.005	1.00E-08	480359
rs11643192	16	A	C	0.027002	0.0049	3.36E-08	480359
rs8063603	16	A	G	-0.0308	0.0053	6.86E-09	480359
rs17727765	17	C	T	0.050799	0.0088	8.51E-09	480359
rs12958048	18	G	A	-0.0338	0.0051	3.61E-11	480359
rs11663393	18	A	G	0.0278	0.0049	1.65E-08	480359
