# Supplementary material for: Noncausal effects of genetic predicted depression and colorectal cancer risk: A Mendelian randomization study
Source: Medicine (Baltimore). 2022 Aug 26;101(34):e30177. doi: 10.1097/MD.0000000000030177 (PMC9410676; doi:10.1097/MD.0000000000030177)
Supplement: Supplementary file 2 [file medi-101-e30177-s002.pdf]

Supplementary Table 2. 41 SNPs associated with MD

SNP	CHR	Effect allele	Other allele	EAF	Beta	SE	P value	Sample size
rs10890030	1	T	C	0.5026	-0.0271	0.0043	3.18E-10	500199
rs4141983	1	C	T	0.326	-0.0264	0.0046	9.69E-09	500199
rs10913112	1	T	C	0.378	-0.0262	0.0045	4.53E-09	500199
rs11579246	1	G	A	0.0914	-0.0491	0.0075	6E-11	500199
rs17641524	1	T	C	0.2101	-0.03	0.0053	1.5E-08	500199
rs7538938	1	C	T	0.5599	0.0251	0.0043	7.29E-09	500199
rs2568958	1	A	G	0.6042	0.0382	0.0044	2.9E-18	500199
rs7551758	1	G	T	0.5329	0.0283	0.0043	5.11E-11	500199
rs72948506	2	A	G	0.2975	0.0265	0.0047	1.71E-08	500199
rs2111592	2	A	G	0.3141	0.0263	0.0046	1.35E-08	500199
rs699927	3	G	T	0.4171	0.024	0.0044	3.79E-08	500199
rs9831648	3	T	G	0.7739	-0.0292	0.0052	1.59E-08	500199
rs66511648	3	C	T	0.284	0.0297	0.0048	6.03E-10	500199
rs843812	3	A	G	0.4117	0.0248	0.0044	1.41E-08	500199
rs7725715	5	A	G	0.5343	0.029	0.0043	1.61E-11	500199
rs30266	5	A	G	0.3271	0.0366	0.0046	1.43E-15	500199
rs247910	5	G	A	0.457	0.0237	0.0043	4.71E-08	500199
rs2214123	6	G	A	0.6466	-0.0261	0.0045	8.56E-09	500199
rs2232423	6	G	A	0.1056	-0.062	0.007	1.14E-18	500199
rs9364755	6	G	A	0.2262	0.0283	0.0051	3.49E-08	500199
rs115938232	6	G	A	0.0599	0.0641	0.0092	3E-12	500199
rs59082935	7	T	C	0.1342	0.0363	0.0066	3.07E-08	500199
rs3807865	7	A	G	0.4105	0.031	0.0044	1.09E-12	500199
rs150346963	7	T	C	0.4118	0.0283	0.0044	1.16E-10	500199

rs10235664	7	C	T	0.2529	-0.027	0.0049	4.68E-08	500199
rs2522831	7	C	T	0.4739	0.024	0.0043	2.11E-08	500199
rs62535714	9	A	G	0.1639	0.0339	0.0058	4.69E-09	500199
rs59283172	9	A	G	0.1081	-0.039	0.007	2.41E-08	500199
rs2418449	9	C	T	0.281	-0.0281	0.0048	4.25E-09	500199
rs1931388	9	G	A	0.4042	-0.0295	0.0044	1.68E-11	500199
rs1021363	10	G	A	0.6434	-0.03	0.0045	2.29E-11	500199
rs198457	11	T	C	0.1886	-0.0315	0.0056	1.9E-08	500199
rs4497414	11	C	T	0.44	0.0291	0.0044	2.93E-11	500199
rs61914045	12	A	G	0.2034	0.0309	0.0054	7.96E-09	500199
rs9529218	13	T	C	0.2031	-0.034	0.0054	2.23E-10	500199
rs508502	13	T	C	0.2992	-0.0264	0.0048	3.56E-08	500199
rs9536381	13	T	C	0.3259	0.0255	0.0046	2.62E-08	500199
rs7152906	14	C	T	0.5196	0.0258	0.0043	1.87E-09	500199
rs1950829	14	G	A	0.5173	-0.0297	0.0043	4.74E-12	500199
rs7241572	18	A	G	0.2047	0.0323	0.0054	2.43E-09	500199
rs17410557	18	C	T	0.3917	0.0267	0.0044	1.42E-09	500199
rs4799949	18	T	C	0.6684	-0.0292	0.0046	1.4E-10	500199
rs4131791	18	T	C	0.4075	-0.0269	0.0044	8.06E-10	500199
rs13037326	20	T	C	0.2597	0.031	0.0049	2.4E-10	500199
