# Supplementary material for: Noncausal effects of genetic predicted depression and colorectal cancer risk: A Mendelian randomization study
Source: Medicine (Baltimore). 2022 Aug 26;101(34):e30177. doi: 10.1097/MD.0000000000030177 (PMC9410676; doi:10.1097/MD.0000000000030177)
Supplement: Supplementary file 3 [file medi-101-e30177-s003.pdf]

Supplement Table 3. Details of the summary data involved in this study

Consortium	Phenotype	Participants	Web source
Meta-analysis	Major depressive disorder	480,359	https://doi.org/10.1038/s41588-018-0090-3
Meta-analysis	Major depression	500,199	https://pubmed.ncbi.nlm.nih.gov/30718901/
FinnGen	Colorectal cancer	218,792	https://finngen.gitbook.io/documentation/
FinnGen	Smoking	138,088	https://finngen.gitbook.io/documentation/
Meta-analysis	Alcohol consumption	939,908	https://pubmed.ncbi.nlm.nih.gov/30643258/
Meta-analysis	Body mass index	339,224	https://pubmed.ncbi.nlm.nih.gov/25673413/
