# Supplementary figures and images for: Noncausal effects of genetic predicted depression and colorectal cancer risk: A Mendelian randomization study
Source: Medicine (Baltimore). 2022 Aug 26;101(34):e30177. doi: 10.1097/MD.0000000000030177 (PMC9410676; doi:10.1097/MD.0000000000030177)

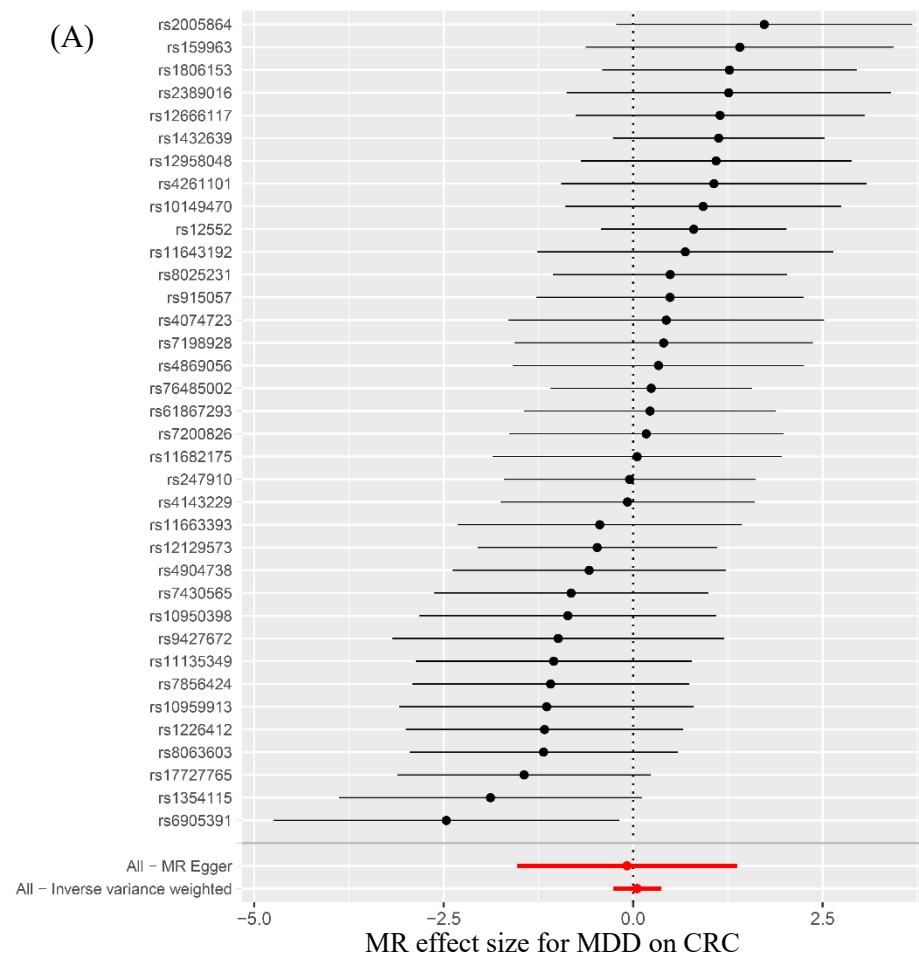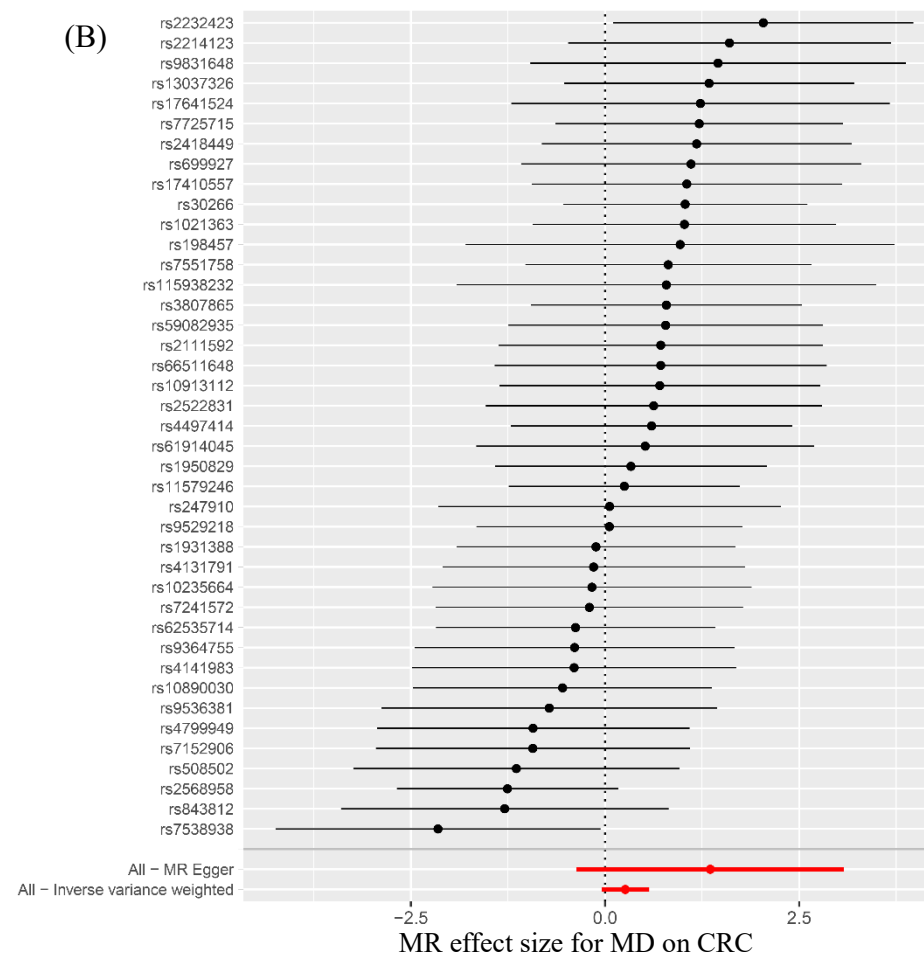

**Supplement Fig. 1** Forest plot of SNPs related to MDD or MD and their CRC risk

Supplement: Supplementary file 4 [file medi-101-e30177-s004.pdf]

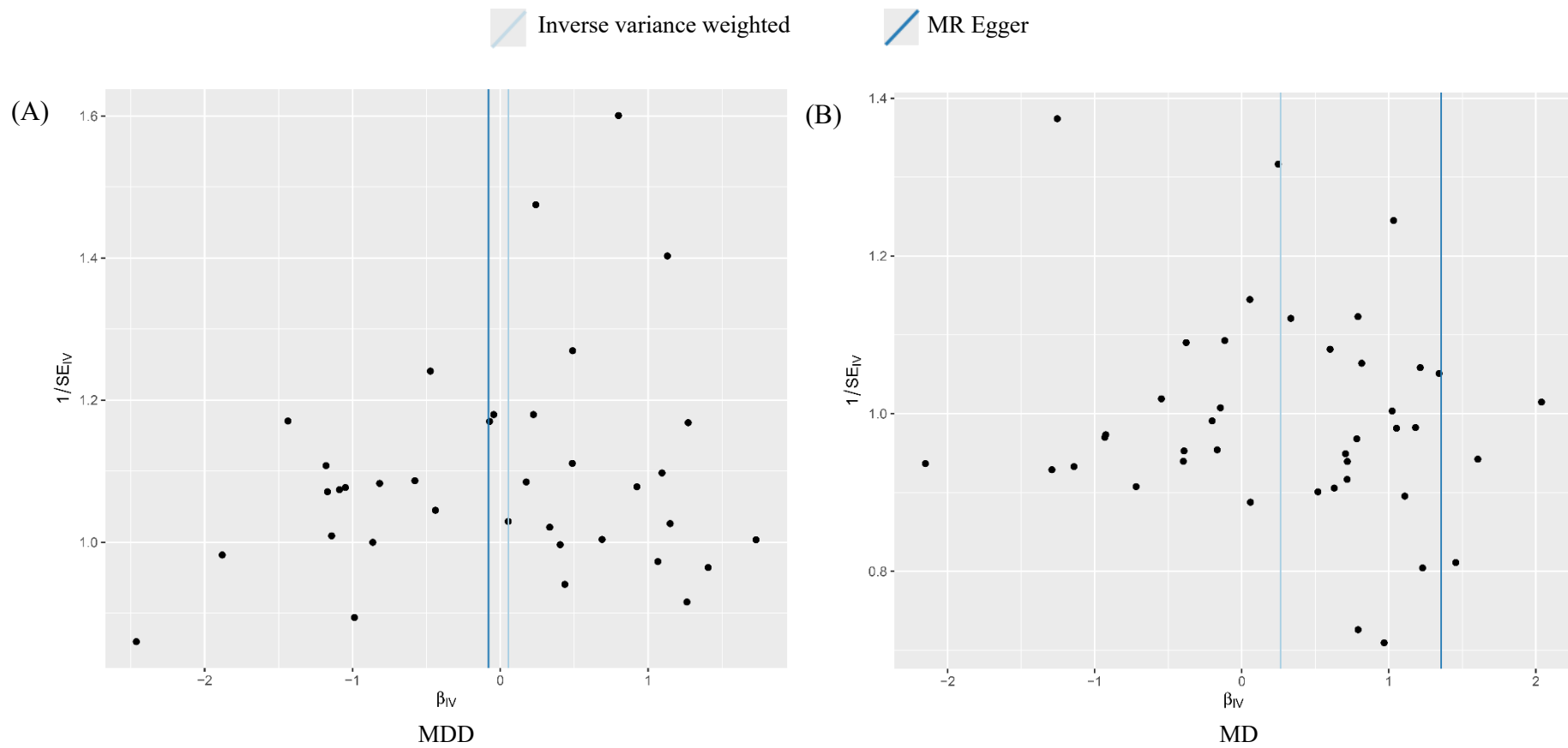

**Supplement Fig. 3** Funnel plot of SNPs associated with MDD or MD and their CRC risk

Supplement: Supplementary file 7 [file medi-101-e30177-s007.pdf]
