# Supplementary material for: Noncausal effects of genetic predicted depression and colorectal cancer risk: A Mendelian randomization study
Source: Medicine (Baltimore). 2022 Aug 26;101(34):e30177. doi: 10.1097/MD.0000000000030177 (PMC9410676; doi:10.1097/MD.0000000000030177)
Supplement: Supplementary file 5 [file medi-101-e30177-s005.pdf]

Supplement Table 4. *P* value of the heterogeneity test and pleiotropy test between exposure and CRC

Exposure	*p* for horizontal pleiotropy [a]	*p* for pleiotropy [b]	*p* for heterogeneity [c]
MDD	0.201	0.855	0.244
MD	0.727	0.214	0.743

Abbreviation: MDD, major depressive disorder; MD, Major depression; MR, Mendelian randomization; [a], MR-PRESSO method was performed to test *p*-value for widespread horizontal pleiotropy; [b], we used the *p*-value from the MR-Egger regression intercept to test the pleiotropy, and [c], performed Cochran's Q test to examine the heterogeneity.
