# Supplementary material for: Noncausal effects of genetic predicted depression and colorectal cancer risk: A Mendelian randomization study
Source: Medicine (Baltimore). 2022 Aug 26;101(34):e30177. doi: 10.1097/MD.0000000000030177 (PMC9410676; doi:10.1097/MD.0000000000030177)
Supplement: Supplementary file 6 [file medi-101-e30177-s006.pdf]

(A)

(B)

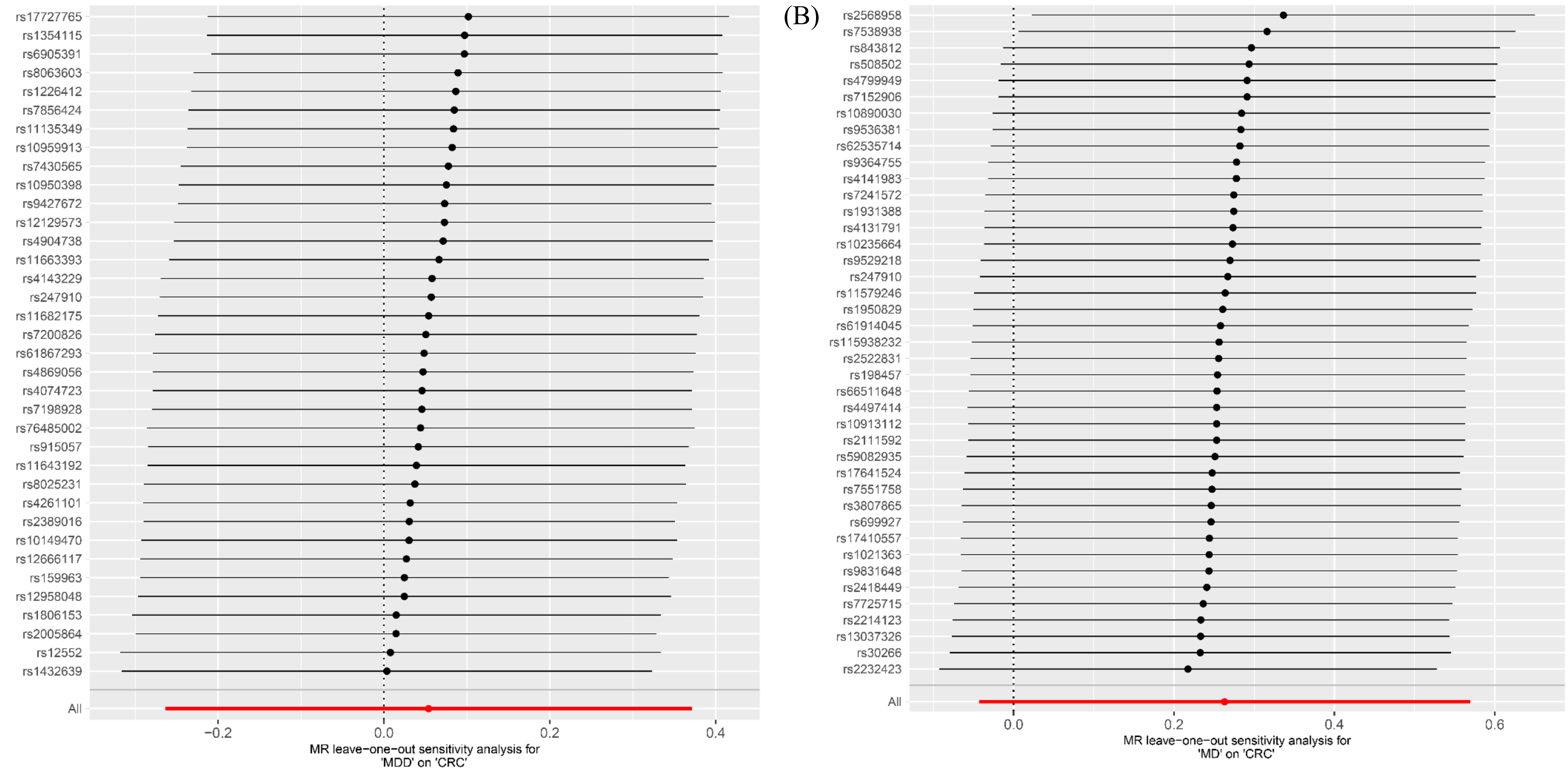

Supplement Fig. 2 Leave-one-out sensitivity test of SNPs associated with MDD or MD and their CRC risk
